# Clinical characteristics and outcome predictors of a Chinese childhood-onset myasthenia gravis cohort

**DOI:** 10.3389/fped.2022.996213

**Published:** 2022-09-29

**Authors:** Lifen Yang, Yulin Tang, Fang He, Ciliu Zhang, Miriam Kessi, Jing Peng, Fei Yin

**Affiliations:** Department of Pediatrics, Xiangya Hospital, Central South University, Changsha, China

**Keywords:** myasthenia gravis, child-hood onset, clinical characteristics, outcome, Chinese

## Abstract

Myasthenia gravis is an organ-specific autoimmune disease. Currently there is no universal guidelines for childhood-onset myasthenia gravis, therefore, treatment strategies are usually based on the guidelines from adult myasthenia gravis patients. In order to contribute in the process of the development of the universal childhood-onset myasthenia gravis guideline, we have summarized the clinical characteristics, treatment strategies, outcome and the related predictors of childhood-onset myasthenia gravis. We recruited 343 childhood-onset myasthenia gravis cases who were followed up at the Department of Pediatrics, Xiangya Hospital from June, 2010 to December, 2019. The data about clinical characteristics, treatments and outcome were collected and analyzed. Among of the 343 cases, 164 cases were followed up for longer than 2 years, of whom 142 still remained with ocular myasthenia gravis at the endpoint. About the treatments, 27 cases (27/164) accepted pyridostigmine only while the rest accepted glucocorticoid and/or other immunosuppressants. At the endpoint, the proportion of optimal outcome was 66.2% in the group remaining with ocular myasthenia gravis and 31.8% in the generalized myasthenia gravis group. Multivariate logistic regression analysis revealed that generalized myasthenia gravis type and positive status of antibodies against acetylcholine receptors were the independent risk factors for poor outcome. In conclusion, our childhood-onset myasthenia gravis patients present mainly as ocular myasthenia gravis, adequate immunotherapy improve the long-term outcome, and generalized myasthenia gravis phenotype as well as positive status of antibodies against acetylcholine receptors relate to poor outcome.

## Introduction

Myasthenia gravis (MG) is an autoimmune disease mediated by antibodies against acetylcholine receptors (AChR) at the endplates or other related postsynaptic molecules involved in neuromuscular transmission (1). At present, the pathogenic roles for antibodies against AChR (AChR-Ab) and muscle-specific kinase (MuSK) have been demonstrated (2). The classical manifestation of MG includes fluctuating muscle weakness and fatigability. If the weakness is restricted to the external ocular muscles, it is termed as ocular MG (OMG), otherwise it is called generalized MG (GMG) when the weakness extends to other muscles (1). Childhood-onset MG (CMG) refers to MG that occurs in childhood. The clinical characteristics as well as outcome differs from adult MG (AMG), besides, they vary across different regions and ethnic groups (3–9). In Asia continent especially in China, CMG is estimated to account for around 50% of the whole MG population (6), in other areas, the percentages are lower (3, 5, 10). At the moment, there is no universal guidelines for CMG, therefore, treatment strategies are usually based on the guidelines from adult MG patients. Since children are a special population to whom doctors and guardians worry more about the side effects of corticosteroid or other immunosuppressants, more clinical and basic research data are needed to diagnose and treat CMG patients more precisely.

As far as we know, there have been few reports of CMG cohorts studying clinical characteristics with different conclusions. Gui et al. reported the long-term follow-up results of 424 CMG patients in which they found only 16.7% of the cases improved significantly (6). Huang et al. studied a juvenile MG (JMG) cohort in southern China in which they observed a higher optimal outcome rate (59.6%) (11). In addition, their study revealed AChR-Ab titer as an independent risk factor for generalization (11). Vanikieti et al. reported that in juvenile OMG, ptosis was more responsive to the treatments than duction limitation (5). Vecchio et al. studied a pediatric cohort which included 74 patients and revealed that onset age younger than 10 years, lack of AChR-Ab on radioimmunoprecipitation and normal repetitive nerve stimulation (RNS) at diagnosis were associated with a higher likelihood of drug free remission (4). Here we have summarized and analyzed our data, hoping to find out some biomarkers to help to predict the outcome then precisely choose optimal therapies and achieve a better outcome for CMG patients in the future.

## Materials and methods

This is a retrospective study. It was approved by the ethical committee of Xiangya Hospital, Central South University and was conducted according to the tenets of the Declaration of Helsinki. Clinical information was obtained from medical records. Informed written consents were provided by the parents/guardians of the participants.

All patients recruited were diagnosed with MG before the age of 14 years from June, 2010 to December, 2019 at the Department of Pediatrics, Xiangya Hospital, Central South University, including in- and out-patients. The inclusion criteria were: (1) children who developed symptoms and were diagnosed before the age of 14 years, (2) cases that presented with typical fluctuating weakness and fatigability of the affected muscles, (3) cases with at least one of the following confirmatory tests: (a) unequivocally positive response to intramuscular injection of a bolus of neostigmine sulfate, (b) positive serum AChR or MuSK antibodies, and (c) positive repetitive nerve stimulation (RNS) results (6). Patients diagnosed with alternative diseases such as transient neonatal MG and congenital myasthenic syndrome during the follow-up were excluded. Totally, we recruited 343 CMG patients for clinical characteristics study, of them, 164 cases were followed up for longer than 2 years to analyze the treatments and possible factors that can influence the outcome. We divided the 164 patients into two groups: one group contained patients who remained with OMG (OMG-R) phenotype at the end of follow-up, the other group named GMG group which included cases presented as GMG at onset and the ones who secondarily progressed into GMG (SGMG) during the follow-up.

The AChR and MuSK antibodies were measured using enzyme-linked immunosorbent assay (ELISA), and the titer above 0.45 nmol/L for AChR-antibody was considered positive; while for MuSK-antibody, the titer ≥0.4 U/ml was considered positive according to the manufacturer's instructions (IBL International GmbH, Germany). RNS was performed whenever possible. Thyroid related examinations including T3, T4, thyroid stimulating hormone (TSH), anti-thyroglobulin antibody (TG-Ab), anti-thyroid peroxidase antibody (TPO-Ab) and other connective tissue diseases related autoantibodies were measured in most of the cases. Abnormal levels of T3, T4, TSH, TG-Ab or TPO-Ab were collectively referred as thyroid-related abnormality. During the follow up, we evaluated complete blood count, liver and renal function, muscle enzymes and electrolyte regularly to monitor the side effects when we prescribed glucocorticoid and other immunosuppressants. The thymus status was evaluated *via* chest x-ray or computed tomographic (CT) scan.

The clinical data including age of onset, gender, family history of autoimmune diseases, proceeding events, initial manifestations, duration, complications, autoantibodies status, RNS results, thymus status, clinical types of classification, treatment modalities and outcome were collected. Patients were usually asked to visit pediatric neurologists every 1–2 months at the beginning of treatments, then every 3 months for therapy adjustments when the disease statuses were stable, the visit intervals were extended to 6 months or longer when the patients became symptoms free. Telephone or online communications were employed when the patients became asymptomatic or drugs had been withdrawn for years.

A routine therapeutic regimen was employed to treat CMG in our center. Briefly, all patients were prescribed with oral pyridostigmine (Py) firstly. If the improvement was not satisfactory or even became deteriorated for a few weeks to months, glucocorticoid (GC) was added. If the responses were still poor after an adequate trial of GC, or GC could not be withdrawn due to symptoms relapse or if the use of GC was contraindicated due to the basic condition of the patients, nonsteroidal immunosuppressants (IS) were added. Azathioprine, tacrolimus, mycophenolate mofetil and so on were chosen as immunosuppressants for different patients based on the individual's situation. About the use of GC, two approaches existed in this study. In the early years, for some patients we used intravenous methylprednisolone pulse (IVMP) at the beginning and then switched to oral prednisone, while for the others especially the ones treated within recent years, we prescribed oral prednisone directly. We started with high dose of oral prednisone (0.5–0.75 mg/kg.d), if the response was not satisfactory, the dose was increased to a maximum of 2 mg/kg.d (all less than 60 mg/day in total). After achieving optimal response, we tapered prednisone gradually to a relative low dose which could keep the patients in a stable status. If the symptoms relapsed during the tapering course or after withdraw, we resumed back to the previous dose or restarted the use of GC or added another IS. No thymectomy was performed in this study because doctors and parents were more concerned about the role of the thymus in the development of the immunological system.

Myasthenia Gravis Foundation of America (MGFA) classification was used to evaluate disease severity (12), among of them, class I was referred to ocular MG (OMG), and class II-V were combined as generalized MG (GMG). At the end of the follow-up, the clinical status was determined according to MGFA Post-Intervention Status (MGFA-PIS) (12). Based on the outcome, we grouped patients into: (1) optimal outcome group: including complete stable remission (CSR), pharmacologic remission (PR) and minimal manifestation (MM); (2) poor outcome group: including unstable status (US, consisting of improved, unchanged, worsened and exacerbated) and death (D).

Statistical analysis was performed with SPSS 24.0 software. For comparison between groups, the Student's *t*-test and the *χ*^2^ test or Mann-Whitney *U* test were used, and a *P* < 0.05 was considered to be statistically significant. Whereas, logistic regression analysis was used to evaluate factors that may affect the outcome or generalization.

## Results

### Clinical characteristics of 343 CMG patients

**Figure 1:**
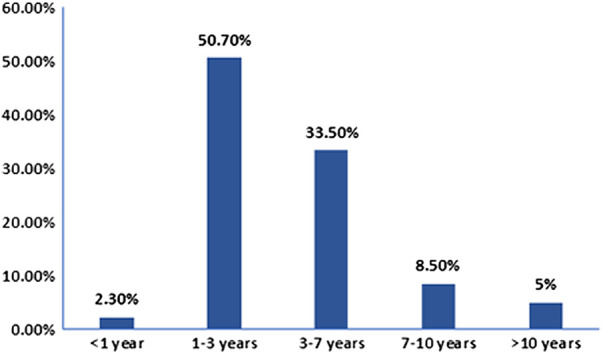
Childhood-onset myasthenia gravis onset age distribution. The number of patients were calculated based on the onset age. Of 343 cases, 174 had onset age between 1 and 3 years, 8 less than 1 year, 115 between 3 and 7 years, 29 between 7 and 10 years, and 17 older than 10 years.

We retrospectively reviewed clinical records of 343 CMG patients. The clinical characteristics are summarized in Table 1. In total 137 males and 206 females were included, the male-to-female ratio was 1:1.5. The median onset age was 34 months (ranged from 3 months to 13 years and 1 month). The onset age distribution is shown in Figure 1; 8 cases (2.3%) had onset age of less than 1 year, 174 cases (50.7%) between 1 and 3 years, 115 cases (33.5%) between 3 and 7 years, 29 cases (8.5%) between 7 and 10 years, while 17 cases (5.0%) after 10 years. Forty-eight cases (48/300, 16%) had thyroid-related abnormalities, among them 6 were diagnosed with hyperthyroidism while 1 with hypothyroidism, 1 with Henoch-Schonlein purpura (HSP) and 1 with type I diabetes. Nineteen cases (5.5%) had autoimmune diseases family history, among of them, 11 with family history of thyroid diseases (hyperthyroidism was the most common), 2 with family history of MG, 1 with family history of SLE, 1 with family history of leukoderma and 1 with family history of allergic rhinitis.

**Table 1:** Clinical characteristics of 343 childhood-onset myasthenia gravis patients.

Characteristic	Patients	Percentage (%)
Gender		
Female	206	60.1
Male	137	39.9
Age at onset		
Younger than 1 year	8	2.3
1–3 years	174	50.7
3–7 years	115	33.5
7–10 years	29	8.5
Older than 10 years	17	5.0
Mean age at onset (months)	45.8 ± 32.5	
Proceeding events (*n* = 305)	60	19.7
Classification at onset		
OMG	330	96.2
GMG	13	3.8
Thyroid-related abnormalities	48/300	16
Autoimmune diseases family history	19	5.5
Neostigmine test (+)	324/337	96.1
Autoantibody		
AChR	82/193	42.5
MuSK	12/180	6.7
Thymus abnormalities		
Hyperplasia	50/321	15.6
Suspected thymoma	6/321	1.9
Abnormal neurophysiology (RNS)	39/96	40.6

OMG, ocular myasthenia gravis; GMG, generalized myasthenia gravis; AChR, acetylcholine receptors; MuSK, muscle-specific kinase; HSP, Henoch-Schonlein purpura.

The preceding events which might had triggered the disease included previous respiratory tract infection (46/305, 15.1%), injuries around the eyes (8/305, 2.6%), vaccinations (5/305, 1.6%), and mosquito bites around the eyes (1/305, 0.3%).

Three hundred and thirty cases (330/343, 96.2%) presented with OMG at onset, including 301 (87.8%) with ptosis only, 12 (3.5%) with diplopia or ocular duction limitation without ptosis, and 17 (5.0%) with combination of ptosis and diplopia or ocular duction limitation. Thirteen cases (3.8%) showed GMG symptoms at onset.

In regards to diagnosis, of the 337 cases who received bolus of neostigmine sulfate intramuscularly, 324 cases (96.1%) showed positive responses. AChR-Abs were detected in 193 cases, 82 cases (42.5%) showed positive results; while 12 cases (12/180, 6.7%) were positive for MuSK antibody tests (including 1 patient with both AChR-Abs and MuSK-Abs at the same time). RNS was carried out in 96 patients and abnormal results were found in 39 cases (40.6%).

Three hundred and twenty-one patients received thymic examination; 20 accepted chest x-ray while 301 underwent CT scan. Of them, 50 cases (15.6%) showed hyperplasia, 6 (1.9%) were suspected to have thymoma, however, during our follow-up period, no thymectomy was performed and no thymoma was confirmed based on histopathology.

### Treatments and outcome of 164 CMG patients with follow-up duration longer than 2 years

One hundred and sixty-four cases were followed up for longer than 2 years. Three patients presented with GMG initially while 161 cases presented with OMG at onset. Of the 161 cases who presented with OMG initially, 142 (86.6%) had OMG-R at the end of follow-up, 19 progressed to secondary generalized myasthenia gravis (SGMG), 10 of them transformed within 2 years after onset.

**Figure 2:**
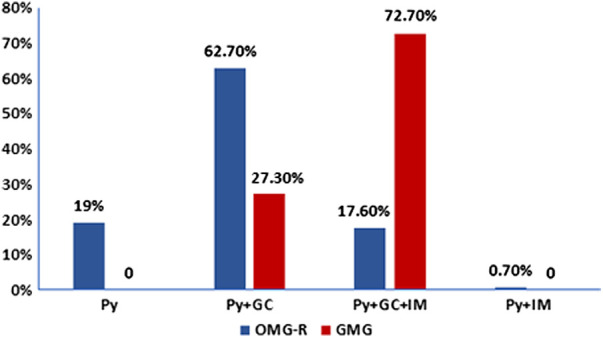
Treatment strategies distribution in remained ocular myasthenia gravis (OMG-R) and generalized myasthenia gravis (GMG) groups. The number of patients who were followed for longer than 2 years in each group (OMG-R or GMG) were calculated based on different treatment strategies. Py, treated with pyridostigmine only; Py + GC: treated with pyridostigmine and glucocorticoid; Py + GC + IM: treated with pyridostigmine, glucocorticoid and other immuosuppressants; Py + IM: treated with pyridostigmine and other immuosuppressants.

About the treatments, as shown in Figure 2, in the OMG-R group, 27 cases accepted Py only, 89 cases accepted the combination of Py + GC, 25 cases accepted Py + GC + IS, and 1 patient accepted Py + IS without GC due to the comorbidity of diabetes. Among the GMG group, 6 and 16 patients accepted the treatments of Py + GC and Py + GC + IS, respectively. Immunosuppressants were more used in the GMG group compared to the OMG-R group (*χ*^2^ = 29.609, *P* = 0.000). We also analyzed the possible long-term effects of different ways of administrating GC. To exclude other interferences, we performed this comparison within OMG-R group accepting Py + GC. Of the 89 cases, 24 cases accepted high-dose IVMP therapy at the beginning then switched to oral prednisone, whereas, 65 cases received oral prednisone directly. At the endpoint, for those who received IVMP, 19 and 5 cases reached optimal and poor outcome respectively, while 46 and 19 cases that received direct oral prednisone reached optimal and poor outcome respectively. There was no difference between the two ways of administrating GC in regards to long-term outcome (*χ*^2^ = 0.628, *P* = 0.428).

**Figure 3:**
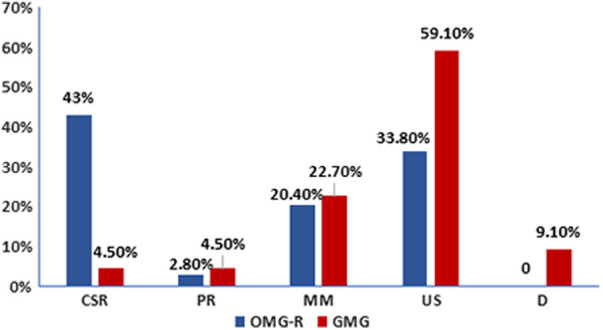
Outcome distribution in remained ocular myasthenia gravis and generalized myasthenia gravis groups. The number of patients who were followed for longer than 2 years in each group (OMG-R or GMG) were calculated based on different outcomes. CSR, complete stable remission; PR, pharmacologic remission; MM, minimal manifestation; US, unstable (including improved, unchanged, worsened and exacerbated); D, death.

With regards to the outcome, among the OMG-R group, 61, 4, 29, 48 cases reached CSR, PR, MM, and US, respectively, while in the GMG group, 1, 1, 5, 13, 2 patients reached CSR, PR, MM, US, D of MG respectively (Figure 3). The optimal outcome (consisting of CSR, PR and MM) proportion was higher in OMG-R group (66.2%) than in GMG group (31.8%) (*χ*^2^ = 9.517, *P* = 0.002).

About the factors that may affect the long-term outcome, as shown in Table 2, we analyzed gender, onset age, proceeding events, disease severity (classified as OMG-R or GMG), AChR-Abs status, abnormal RNS, thyroid-related abnormalities and family history of autoimmune diseases. Univariate logistic regression analysis revealed that there's no difference between the optimal and poor outcome group with regards to gender, onset age, proceeding events, abnormal RNS, thyroid-related abnormalities and family history of autoimmune diseases, but we noticed more OMG-R cases and less AChR-Abs positive cases within the optimal outcome group compared with poor outcome group. When we analyzed these findings with multivariate logistic regression, we found that GMG type (OR = 4.164; 95%CI, 1.419–12.220) and positive AChR-Abs status (above 0.45 nmol/L) (OR = 2.727; 95%CI, 1.209–6.153) were independent risk factors for poor outcome. Abnormal RNS appeared more common in the poor outcome group but without statistical significance. To further investigate the predictive role of RNS, we compared cases with abnormal RNS findings against those with normal RNS findings. There were 21 cases with abnormal RNS findings, and 2 of them reached CSR. Whereas, among 24 cases with normal RNS results, 11 of them achieved CSR, indicating that CSR proportion was significantly higher in the group with normal RNS findings (*χ*^2^ = 7.188, *P* = 0.007).

**Table 2:** Comparison of clinical characteristics between optimal and poor outcome group.

Characteristic	Optimal, *N* (%)	Poor, *N* (%)	*χ*^2^/Z value	*P*–value
Gender				
Male	43 (43)	28 (44)	0.055	0.814
Female	58 (58)	35 (56)		
Age of onset (mean, months)	81.43	84.22	−0.367	0.714
Proceeding events				
Yes	16 (16)	12 (20)	0.368	0.544
No	81 (84)	47 (80)		
Disease severity				
OMG-R	94 (93)	48 (76)	9.517	**0.002** *
GMG	7 (7)	15 (24)		
AChR-Ab (positive)	17 (28)	23 (53)	6.673	**0.010** *
Abnormal RNS	9 (35)	11 (61)	3.012	0.083
Thyroid-related abnormalities	15 (16)	7 (13)		0.654
Autoimmune disease family history	7 (7)	5 (8)	0.201	0.768

OMG-R, remained ocular myasthenia gravis at the end of follow-up; GMG, generalized myasthenia gravis; AChR-Ab, antibody against acetylcholine receptors; RNS, abnormal neurophysiology.

* Significant differences at *P* value <0.05.

During the follow up, we also evaluated the side effects of the treatments. The side effects such as gain of weight and slow growth were very common for the glucocorticoid especially when the dose was above 0.5 mg/kg.d, these effects disappeared soon when glucocorticoid was tapered. Totally we used other IS in 42 patients. Among them, azathioprine was used in 17 cases, tacrolimus in 29 cases, mycophenolate mofetil in 5 cases and cyclosporin in 3 cases. For the side effects of azathioprine, one case was observed with decreased white blood cells (WBC) 9 months after the addition of azathioprine and when it was withdrawn the number of WBC recovered, two cases with decreased hemoglobin, one other case was found to have abnormal glutamic-pyruvic transaminase (ALT) and creatine kinase (CK), these changes were transient and recovered automatically which didn't lead to the withdraw of the drug. For tacrolimus, increased uric acid in 6 cases, increased CK in 5 cases, increased ALT in 1 case and hypomagnesemia in 2 cases were observed-all the abnormal parameters were slight as well as transient and recovered automatically which didn't lead to the withdraw of the drug. For the few cases prescribed with mycophenolate mofetil and cyclosporin, no obvious abnormalities were observed. So during the treatment with immunosuppressants, the routine side effects of glucocorticoid were definite and common although disappeared soon when it was tapered, for other immunosuppressants, we only withdrew azathioprine in one case due to decreased WBC but we didn't see severe side effects. Basically, we think our patients were tolerant with these immunosuppressants.

### Transformation of OMG to GMG

At the end of the follow-up, 19 out of the 161 cases progressed into SGMG. The median duration from onset to progression to SGMG was 68 months; for 52.6% of the cases it was less than 2 years while for 15.8% of the cases it was longer than 5 years. We tried to find any factors that may affect this transformation. Univariate logistic regression analysis revealed none of the factors including gender, age of onset, follow-up duration, proceeding events, onset manifestation, AChR-Abs status, abnormal RNS and thyroid-related abnormalities were closely associated with the progression (Table 3).

**Table 3:** Comparison of clinical characteristics between cases that remained with ocular myasthenia gravis with secondary generalized myasthenia gravis.

	SGMG	OMG-R	*χ*^2^/Z value	*P*-value
Gender				
Male	5 (26)	64 (45)	2.407	0.121
Female	14 (74)	78 (55)		
Age of onset (mean, months)	82.7	81.2	−0.138	0.891
Follow-up duration (mean, months)	81.5	88.8	−0.671	0.502
Proceeding events				
Yes	3 (16)	24 (18)		1.000
No	16 (84)	110 (82)		
Onset manifestation				
Ptosis	17 (89)	129 (95)	0.897	0.304
Ptosis and other ocular manifestation	2 (11)	7 (5)		
AChR-Ab (positive)	9 (50)	35 (38)		0.344
Abnormal RNS	3 (60)	17 (59)		0.646
Thyroid-related abnormalities	4 (24)	17 (13)		0.276

SGMG, secondary generalized myasthenia gravis; OMG-R, remained ocular myasthenia gravis at the end of follow-up; AChR-Ab, antibody against acetylcholine receptors; RNS, abnormal neurophysiology.

## Discussion

According to previous studies, CMG accounts for around half of the total MG population in China, and there is still no universal treatment guideline for this special group (6, 11). A few studies of CMG patients have reported the predictors for relapse as well as generalization, nevertheless, some of the conclusions are controversial. It requires more clinical and basic researches to identify the prognostic factors for outcome as well as generalization that can guide in the provision of the appropriate treatment regimens which can control the symptoms well with tolerable side effects at the same time.

In this study, we have described clinical characteristics of our 343 CMG patients, it is one of the biggest CMG cohorts reported yet. Previous studies in China showed that CMG manifested mainly before puberty, the most common onset symptom was ptosis, and the rate of generalization of CMG was pretty low (less than 20%) (6, 11, 13). In our study the median onset age was 34 months with a peak between 1 and 7 years (84.2% in total), the male-to-female ratio was 1:1.5 showing a minor female predominance. Consistent with previous studies (6, 11), we found that 96.2% of the patients presented with ocular symptoms only at onset, ptosis was the most common symptom, and 86.6% remained with OMG at the end of point when we followed up 164 cases for longer than 2 years. Similar to previous study (13), thyroid-related abnormalities and family history of autoimmune diseases were common in our study, indicating thyroid function as well as the related autoantibodies should be examined routinely for CMG patients.

Previous studies from China reported an AChR-Abs positivity rate between 40% and 78% (6, 13), while this rate was relatively higher in other countries (8, 14). In our current study, the positivity rate of AChR-Abs was 42.5%. One reason for the relatively low rate is that we had a high proportion of OMG, the other reason is that we detected it with ELISA which is less sensitive compared to cell-based assay (CBA) (10). We found 12 positive cases for MuSK-Abs, since this figure was too small and the clinical situations varied we didn't analyze these patients. The rate of thymus hyperplasia was only 15.6% in our cohort, lower than the published one (6), and we couldn't confirm a thymoma with histopathology.

The treatment strategies for CMG patients vary among different regions and ethnics groups, patients within the CMG entity are more reluctant to take immunosuppressants. With regarding to side effects, we observed definite and common side effects for GC, but other IS seemed safe and well-tolerated in our study indicating other immunosuppressants might be considered earlier than we have been doing previously. Gui et al. reported a significant improvement rate of only 16.7% in their CMG cohort (6), while Huang et al. claimed an optimal outcome rate was achieved to 59.6% in a Juvenile MG (JMG) cohort (11). The differences were observed probably due to the different usages of immunotherapy and the duration of follow-up because some patients got relapses after the so-called CSR for longer than 1 year (6). We analyzed the treatments and outcome in 164 patients with longer than 2 years of follow-up. Twenty-seven of them responded well to pyridostigmine only, the others including the OMG-R ones needed GC and/or other IS to achieve a satisfactory status. A few studies reported the efficacy of IVMP in improving ocular symptoms (15, 16). We also gave IVMP as a first therapy in the early years and we found that the choice of glucocorticoids either IVMP first or direct oral prednisone made no difference with regard to the long-term outcome of OMG-R cases, consistent with our previous study (17). Since giving IVMP at the beginning may lead to exacerbation of the condition even MG crisis (18), we gave up this way of giving glucocorticoid in recent years. Although GC and IS were used more common in GMG group than in OMG-R group, the optimal outcome rate in the GMG group was lower, indicating that disease severity was a prognostic factor for the outcome. GMG predicted poorer outcome and required more progressive immunosuppressive treatments earlier in our CMG. When we divided patients into optimal and poor outcome groups to explore the possible prognostic factors, we found that GMG type and positive AChR-Abs status were independent predictors for poor outcome. Vecchio et al. reported that AChR antibodies status was a significant predictor for drug free remission (4), Huang et al. revealed AChR-Ab titer was an independent risk factor for generalization (11), both are consistent with our results in a way. We also noticed that normal RNS predicted a higher possibility of CSR, due to the small sample size, further investigations will be needed to evaluate its role in predicting the long-term outcome of CMG.

Within the 161 initial OMG patients, 11.8% progressed into SGMG, this figure is also consistent with previous report from China (6). Wang et al. reported that thymic hyperplasia could predict generalization within the first 6 months (19). Huang et al. found that AChR-Ab titer was an independent risk factor for generalization (11), however, when we used logistic regression to analyze the possible factors that may contribute to this generalization, we could not find any. One possible reason may be that the size of the transformed population was too small.

It should be pointed out that there are several limitations in this study. It is a retrospective study and done within one hospital so bias may exist. For some patients the antibodies were measured with positive results not at the onset while post-immune therapy, and we didn't recheck the level of the antibodies from time to time, so we didn't evaluate the antibody titer related effects. In comparison to other centers, none of our patients received thymectomy suggesting that doctors in our center are more reluctant to perform such kind of surgery in CMG patients. Notably, this may lead to the loss of data which could be used to evaluate the therapeutic effects of thymectomy in CMG. At last, the number of patients using other immunosuppressants was too small, which made it difficult to compare the therapeutic as well as side effects between the individual drug. Longer follow-up as well as multi-center studies may provide further insights into the clinical characteristics of this special MG entity, finding out the useful prognostic factors which could be used as determinants in choosing immunotherapy.

## Conclusion

In conclusion, our data confirmed that the peak age of CMG in China is pre-puberty, most of the cases remain with the OMG type, and the transformation proportion of OMG into GMG is low. GMG type as well as positive AChR-Ab status predict relatively poor outcome and may require more aggressive treatments. Most of our CMG patients need GC or together with IS treatment to achieve optimal outcome.

## Data Availability

The raw data supporting the conclusions of this article will be made available by the authors, without undue reservation.

## References

[1] Gilhus NE Verschuuren JJ. Myasthenia gravis: subgroup classification and therapeutic strategies. Lancet Neurol. (2015) 14(10):1023–36. 10.1016/S1474-4422(15)00145-3 26376969

[2] Gilhus NE. Myasthenia Gravis. N Engl J Med. (2016) 375(26):2570–81. 10.1056/NEJMra1602678 28029925

[3] Popperud TH Boldingh MI Rasmussen M Kerty E. Juvenile myasthenia gravis in Norway: clinical characteristics, treatment, and long-term outcome in a nationwide population-based cohort. Eur J Paediatr Neurol. (2017) 21(5):707–14. 10.1016/j.ejpn.2017.04.003 28457757

[4] Vecchio D Ramdas S Munot P Pitt M Beeson D Knight R Paediatric myasthenia gravis: prognostic factors for drug free remission. Neuromuscul Disord. (2020) 30(2):120–7. 10.1016/j.nmd.2019.11.008 32001147

[5] Vanikieti K Lowwongngam K Padungkiatsagul T Visudtibhan A Poonyathalang A. Juvenile ocular myasthenia gravis: presentation and outcome of a large cohort. Pediatr Neurol. (2018) 87:36–41. 10.1016/j.pediatrneurol.2018.06.007 30197221

[6] Gui M Luo X Lin J Li Y Zhang M Zhang X Long-term outcome of 424 childhood-onset myasthenia gravis patients. J Neurol. (2015) 262(4):823–30. 10.1007/s00415-015-7638-2 25588729

[7] Chiang LM Darras BT Kang PB. Juvenile myasthenia gravis. Muscle Nerve. (2009) 39(4):423–31. 10.1002/mus.21195 19229875

[8] Lee HN Kang HC Lee JS Kim HD Shin HY Kim SM Juvenile myasthenia gravis in Korea: subgroup analysis according to sex and onset age. J Child Neurol. (2016) 31(14):1561–8. 10.1177/0883073816666206 27581849

[9] Takahashi Y Sugiyama M Ueda Y Itoh T Yagyu K Shiraishi H Childhood-onset anti-MuSK antibody positive myasthenia gravis demonstrates a distinct clinical course. Brain Dev. (2012) 34(9):784–6. 10.1016/j.braindev.2011.12.014 22277190

[10] Barraud C Desguerre I Barnerias C Gitiaux C Boulay C Chabrol B. Clinical features and evolution of juvenile myasthenia gravis in a French cohort. Muscle Nerve. (2018) 57(4):603–9. 10.1002/mus.25965 28877546

[11] Huang X Li Y Feng H Chen P Liu W. Clinical characteristics of juvenile myasthenia gravis in Southern China. Front Neurol. (2018) 9:77. 10.3389/fneur.2018.00077 29535672 PMC5835068

[12] Jaretzki A 3rd Barohn RJ Ernstoff RM Kaminski HJ Keesey JC Penn AS Myasthenia gravis: recommendations for clinical research standards. Task force of the medical scientific advisory board of the myasthenia gravis foundation of America. Neurology. (2000) 55(1):16–23. 10.1212/WNL.55.1.16 10891897

[13] Yang ZX Xu KL Xiong H. Clinical characteristics and therapeutic evaluation of childhood myasthenia gravis. Exp Ther Med. (2015) 9(4):1363–8. 10.3892/etm.2015.2256 25780436 PMC4353784

[14] Melbourne Chambers R Forrester S Gray R Tapper J Trotman H. Myasthenia gravis in Jamaican children: a 12-year institutional review. Paediatr Int Child Health. (2012) 32(1):47–50. 10.1179/1465328111Y.0000000042 22525448

[15] Ozawa Y Uzawa A Kanai T Oda F Yasuda M Kawaguchi N Efficacy of high-dose intravenous methylprednisolone therapy for ocular myasthenia gravis. J Neurol Sci. (2019) 402:12–5. 10.1016/j.jns.2019.05.003 31100651

[16] Lindberg C Andersen O Lefvert AK. Treatment of myasthenia gravis with methylprednisolone pulse: a double blind study. Acta Neurol Scand. (1998) 97(6):370–3. 10.1111/j.1600-0404.1998.tb05968.x 9669469

[17] Liu C Wang X Xie L Peng J Wu L Zheng X. [Analysis for the effect of different regimens on ocular myasthenia gravis in children]. Zhong Nan Da Xue Xue Bao Yi Xue Ban. (2017) 42(11):1275–9. 10.11817/j.issn.1672-7347.2017.11.006 29187654

[18] Sugimoto T Ochi K Ishikawa R Tazuma T Hayashi M Mine N Initial deterioration and intravenous methylprednisolone therapy in patients with myasthenia gravis. J Neurol Sci. (2020) 412:116740. 10.1016/j.jns.2020.116740 32145521

[19] Wang L Zhang Y He M. Clinical predictors for the prognosis of myasthenia gravis. BMC Neurol. (2017) 17(1):77. 10.1186/s12883-017-0857-7 28420327 PMC5395963

